# Effect of latanoprost and timolol on the histopathology of the human conjunctiva

**DOI:** 10.1136/bjo.2008.140186

**Published:** 2008-10-29

**Authors:** N Terai, U Schlötzer-Schrehardt, J Lampel, A G Böhm, C Rummelt, E Schmidt, L E Pillunat

**Affiliations:** 1 Department of Ophthalmology, Carl Gustav Carus University Hospital, Dresden, Germany; 2 Department of Ophthalmology, University of Erlangen-Nuernberg, Erlangen, Germany

## Abstract

**Aim:**

To investigate the effect of timolol and latanoprost on the extracellular matrix organisation, inflammatory infiltration, and expression of matrix metalloproteinases (MMPs) and tissue inhibitors of matrix metalloproteinases (TIMPs) in the human conjunctiva.

**Methods:**

Conjunctival biopsies were obtained from the inferior fornix during routine cataract surgery from 20 patients with primary open-angle glaucoma, who had received a monotherapy either with timolol or latanoprost, and from 10 non-glaucomatous patients. Specimens were investigated by light microscopy, immunohistochemistry using antibodies against MMP-1,-3, TIMP-2,-3 and CD 68 antibodies and by quantitative transmission electron microscopy.

**Results:**

The number of collagen fibres was significantly decreased in latanoprost-treated conjunctival specimens compared with timolol-treated eyes (p*<*0.01) but showed no difference to controls. Amorphous material was increased in both treated groups compared with controls (p<0.001) but was less in latanoprost-treated specimens compared with timolol-treated eyes (p<0.001). Optically clear spaces, probably containing glycosaminoglycans, were significantly reduced in both treated groups—with less of a reduction in latanoprost—compared with timolol-treated eyes (p<0.001). A marked upregulation of MMP-1 and MMP-3 and moderately increased staining for TIMP-2 and TIMP-3 was found in epithelial cells and subepithelial stromal cells of latanoprost-treated eyes. A moderate infiltration with macrophages and inflammatory cells was observed in timolol-treated eyes.

**Conclusions:**

Latanoprost-treated conjunctival specimens showed a decreased stromal collagen density and a less pronounced inflammatory infiltration. The upregulation of MMP-1 and MMP-3 in latanoprost-treated eyes might explain the reduced extracellular matrix accumulation in the conjunctival stroma. Therefore, latanoprost therapy might have a more favourable effect on the outcome of glaucoma filtering surgery.

Antiglaucoma drug medication is the major treatment modality for glaucoma. There is increasing evidence that the long-term use of topically administered medication affects the structure and integrity of the conjunctival tissue and the ocular surface.^1^^–^8

On the other hand glaucoma filtering surgery frequently fails mostly due to fibrosis at the episcleral-, conjunctival/Tenon capsule interface.^9^ Several studies have already suspected a link between an altered histomorphology of conjunctival tissue due to long-term antiglaucoma medication and the risk of filtering failure in patients who had undergone trabeculectomy.^10^ Sherwood *et al* suggested that chronic topical antiglaucoma medication increased the likelihood of fibrosis and subsequent bleb failure.^11^

A previous study investigating the long-term effect of two commonly used antiglaucoma medications, latanoprost and timolol, on the rabbit conjunctiva demonstrated significant differences regarding extracellular matrix composition in the conjunctival stroma between both medications.^12^ Additionally, an upregulation of specific matrix metalloproteinases in the latanoprost treated specimens was detected.

In the present study, we investigated the long-term effect of both antiglaucoma medications on the human conjunctiva. In particular, we analysed the extracellular matrix organisation, inflammatory infiltration and expression of matrix metalloproteinases (MMPs) and their inhibitors (TIMPs) using immunhistochemistry and electron microscopy.

## MATERIAL AND METHODS

### Patients

Based on a statistical power calculation estimating the number of collagen fibres, the amount of amorphous material and empty spaces in the 30 subepithelial layer participants were included in this study. For this estimation, α = 0.05 and β = 0.20 (power of 80%). According to the equation n = 7.85×(SD/difference of the means)^2^, the required number of participants for the parameter collagen fibres (SD 4.4 AU, difference of the means 4.0 AU) was n = 10, for the parameter amorphous material (SD 3.9 AU, difference of the means 4.8 AU) n = 6 and for the parameter empty spaces (SD = 6.3 AU, difference of the means 17.0 AU) n = 3. The study protocol was approved by the ethics committee of Dresden following the declaration of Helsinki. All subjects were recruited from the Department of Ophthalmology (University of Dresden). All subjects signed an informed consent before participating in this trial. Patients were divided into three groups according to the type of topical therapy administered (table 1).

**Table 1:** Clinical data

Group	No of subjects (n)	Mean age (years)	Concentration(mg/ml)	Applications per day	Preservative	Concentration (mg/ml) of benzalkonium chloride
Control	10	76 (6)	–	–	–	–
Timolol	10	75 (7)	5.0	Twice	BAC	0.1
Latanoprost	10	74 (10)	0.05	Once	BAC	0.2

The inclusion criteria for this study were as follows: patients with primary-open angle glaucoma or ocular hypertension treated with a monotherapy of timolol 0.5% (n = 10, mean age 75 (7) years) (Tim-ophthal 5 mg/ml, Novartis) or 0.005% latanoprost (n = 10, mean age 74 (10) years), (Xalatan 50 μg/ml, Pharmacia, Karlsruhe, Germany) for a period of at least 15 months and normal subjects without glaucoma receiving no topical medication (n = 10, mean age 76 (6) years). Intraocular pressure (IOP) measurements in normal subjects were 22 mm Hg or less, with no history of increased IOP. Optic discs were considered normal if they had intact rims, no haemorrhages, notches, excavation, nerve fibre defects, or asymmetry of the vertical C/D ratio more than 0.2. In ocular hypertensive patients, IOP measurements were 23 mm Hg or greater on at least two occasions. Inclusion criteria for the optic disc were the same as in the normal group. Patients with primary open-angle glaucoma were included only if their optic discs were glaucomatous based on neuroretinal rim thinning, notching, excavation, nerve fibre layer defect or asymmetry of the vertical C/D ratio more than 0.2 between both eyes. Visual field and IOP were not used to define glaucoma.

Exclusion criteria were: inflammatory diseases of the eye, the intake of medication affecting wound healing (e. g steroids or immunosuppressors), participants younger than 18 years, scarring diseases of the mucosa and other disorders of wound healing.

After informed consent, 2×2 mm conjunctival biopsies from the inferior fornix of the conjunctiva were obtained during routine cataract surgery from the 20 glaucoma patients and from the 10 non-glaucomatous subjects. The specimens were bisected, immediately immersed in the appropriate fixative and transferred to the Department of Ophthalmology, University of Erlangen-Nuernberg, for morphological and immunohistochemical analysis.

### Methods

#### Immunohistochemistry

The first halves of the specimens were fixed in 4% paraformaldhyde in PBS and embedded in paraffin according to standard protocols. Light-microscopic immunohistochemistry was performed on 5 μm thick paraffin sections using the peroxidase labelled streptavidin-biotin method (LSAB Plus kit; Dako, Glostrup, Denmark) according to the manufacturer’s instructions. Briefly, sections were incubated for 30 min each with monoclonal mouse anti-rabbit MMP-3 (stromelysin), monoclonal mouse anti-rabbit MMP-1, mouse anti-TIMP-2, anti-TIMP-3 and anti-CD 68 antibodies (Chemicon, Temecula, California) at a concentration of 5 μg/ml, with the biotinylated link antibody and horseradish peroxidase (HRP)-conjugated streptavidin. Proteolytic predigestion using proteinase K was performed for 6 min. 3-Amino 9-ethyl carbazole was used as a chromogenic substrate and Mayer haemalum as a counterstain. In negative control experiments, the primary antibody was omitted or replaced by equimolar concentrations of preimmune mouse immunoglobulin G.

#### Transmission electron microscopy

The second halves of the specimens were fixed in 2% glutaraldehyde in PBS and further processed by postfixation in 2% buffered osmium tetroxide and embedded in epoxy resin (Epon 812; Fluka, Buchs, Switzerland) according to standard protocols.

Semithin sections were stained with Toluidine Blue, and ultrathin sections were stained with uranyl acetate and lead citrate and examined with a transmission electron microscope (EM 906E; Leo, Oberkochen, Germany).

For quantitative analysis of extracellular parameters (collagen fibres, amorphous substance, electron-lucent spaces), an automated image-processing system (DigiVision; Leo) with an integrated software package (Analysis; Soft Imaging Systems, Muenster, Germany) was used. Measurements were performed according to a defined random sampling procedure, using the bars of the supporting grid square as points of reference, by which 10 consecutive areas adjacent to the right side of a grid bar were analysed. Ten measurements per specimen were performed in the subepithelial stroma and 10 in the deeper stroma at a distance of approximately 100 μm from the epithelial basement membrane. Only areas including extracellular matrix but avoiding areas with cells or blood vessels were examined. The size of the area analysed was 66.8 μm^2^ at a magnification of 4000×. The different grey values of the structural parameters measured (collagen fibres, amorphous material, empty spaces) were transformed into false colours (red, green and white), and the percentage areas occupied by the different colour-coded phases were automatically calculated.

For statistical analysis of the automatically measured areas, the mean (SD) of the individual 10 measurements was calculated for each layer and tissue component. These mean values were then compared between the control and the treated groups regarding each different component. For this purpose, the non-parametric Mann–Whitney test was used. A p value of <0.05 was considered statistically significant.

## RESULTS

### Light microscopy

In the control group, the conjunctiva consisted of a normal goblet-cell-containing epithelium with an intact epithelial basement membrane, loose collagenous connective tissue, and no acute or chronic inflammation in the substantia propria. In semithin sections, an increase in the density of the collagen fibres in the substantia propria was present in eyes treated with timolol but not in eyes treated with latanoprost.

### Immunohistochemistry

#### MMPs

**Figure 1:**
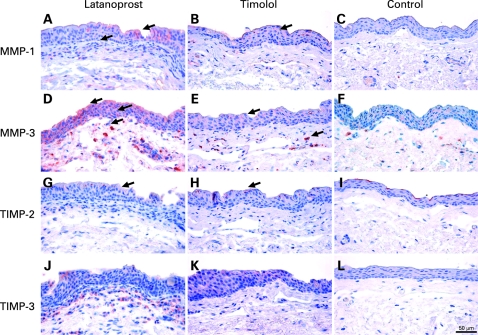
Immunohistochemical staining of MMP-1 (A–C) and MMP-3 (D–F), TIMP-2 (G–I) and TIMP-3 (J–L) in the conjunctival specimens of control and study groups.

In the control group no staining with either the MMP-1 or MMP-3 antibodies was observed, either in the epithelium or in the stroma of the conjunctival specimens (fig 1C,F). In latanoprost-treated conjunctival tissue, a marked upregulation of MMP-1 and MMP-3 was found in the epithelial cells as well as in the subepithelial stroma cells but not in the extracellular matrix (fig 1A). In the timolol-treated specimens, immunoreactions were less pronounced, revealing a very weak positive reaction of staining in the epithelium and the stroma (fig 1B,E).

#### TIMPs

Staining with antibodies to TIMP-1 and TIMP-3 was negative in the control group (fig 1I,L). The latanoprost-treated specimens showed moderately increased staining for TIMP-2 and TIMP-3 in the epithelial cells as well as in subepithelial stroma cells. The timolol-treated eyes revealed a very weak staining with TIMP-1 or TIMP-2 in the epithelium. Similar to the MMP-1 and MMP-3 pattern, TIMPs were immunolocalised to cells but not to the extracellular matrix.

#### CD-68

**Figure 2:**
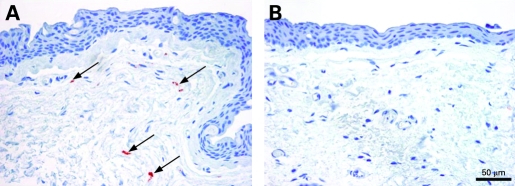
Immunolocalisation of CD 68 indicating macrophages in the conjunctival tissue of eyes treated with timolol (A) and latanoprost (B).

A moderate infiltration with CD-68-positive macrophages was observed in timolol-treated specimens only (fig 2A), whereas no inflammatory reaction was observed in the latanoprost treated group (fig 2B) or in the control group (not shown).

### Quantitative electron microscopy

**Figure 3:**
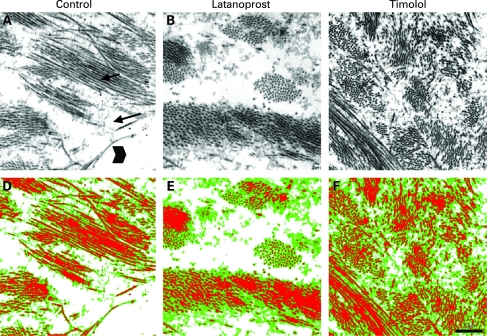
Quantification analysis of extracellular matrix components in the conjunctival stroma: (A–C) electron micrograph showing collagen fibres (small arrow), amorphous material (large arrow), and optically clear spaces (arrowhead) in the control, timolol and latanoprost group: (D–F), original magnification 34 000×.

According to the different intensity of grey values, the three extracellular components collagen fibres, amorphous material and optically clear spaces were coded by false colours red, green and white (fig 3). The area occupied by each colour was automatically measured, and the percentage of each area in relation to the whole field was calculated (table 2).

**Table 2:** Analysis of extracellular conjunctival tissue changes: area occupied by collagen fibres, amorphous material and empty spaces in the subepithelial and deep layer in the control-, timolol- and latanoprost group

Group	Collagen fibres	Amorphous material	Empty spaces
Subepithelial layer			
Control	40.3 (15.3)	30.9 (12.8)	28.8 (20.2)
Timolol	43.3 (17.4) (p>0.05)	43.3 (13.2) (p<0.001)	13.4 (9.5) (p<0.001)
Latanoprost	42.9 (13.2) (p>0.05)	38.1 (10.1) (p<0.001)	19.0 (10.5) (p<0.01)
Deep layer			
Control	33.5 (14.6)	26.6 (12.2)	39.9 (20.9)
Timolol	36.6 (13.2) (p>0.05)	35.1 (9.8) (p<0.001)	32.8 (15.1) (p<0.05)
Latanoprost	31.8 (10.4) (p>0.05)	31.1 (10.4) (p<0.001)	32.7 (14.9) (p<0.05)

Data are presented as mean percentage (SD) (p values) relative to control group are indicated below.

Conjunctival specimens of the control and latanoprost group revealed a loose arrangement of extracellular matrix components with a moderate collagen density and enlarged empty spaces. Amorphous material was only focally present in latanoprost treated eyes (fig 3A,B,D,E).

In contrast, in the timolol group collagen fibres appeared augmented in the deep stroma relative to latanoprost, amorphous material in both the subepithelial and deeper stromal layers relative to the latanoprost and control group (fig 3,C,F).

### Collagen fibres

**Figure 4:**
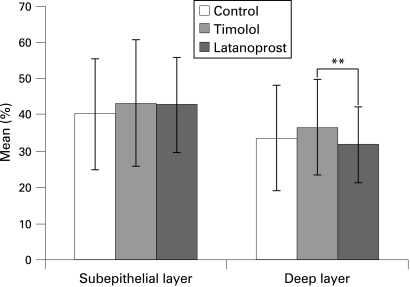
Mean percentage (%) of collagen fibres in the subepithelial and deep layer in the control group (white column), timolol group (grey column) and latanoprost group (black column); *p*<*0.05, **p*<*0.01, ***p*<*0.001.

In the deeper stroma, the mean percentage of area occupied by collagen fibres was significantly decreased in latanoprost-treated specimens (31.8%) as compared with timolol-treated eyes (36.6%) (p<0.01, fig 4). The mean percentage area in the control group was 33.5%, which showed no significant difference compared with the other groups (p>0.05). In the subepithelial layer, no significant difference in the area occupied by collagen could be observed between the three groups (p>0.05, table 2).

### Amorphous material

**Figure 5:**
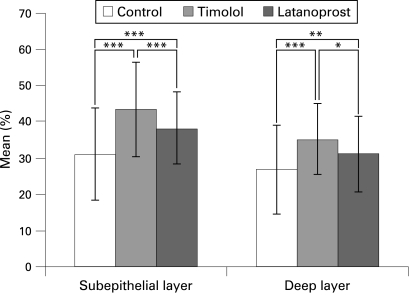
Mean percentage (%) of amorphous material in the subepithelial and deep layer in the control (white column), timolol (grey column) and latanoprost group (black column); *p*<*0.05, **p*<*0.01, ***p*<*0.001.

In both the subepithelial and deeper stroma, the amount of amorphous material was increased in both treated groups as compared with controls (31% and 27%, p<0.001) but the difference was less pronounced in latanoprost-treated specimens (38% and 31%) as compared with timolol-treated eyes (43% and 35%) (fig 5, table 2).

### Empty spaces

**Figure 6:**
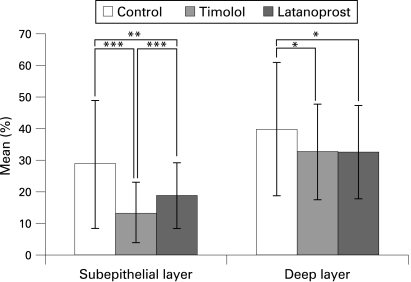
Mean percentage (%) of empty spaces in the subepithelial and deep layer in the control-, (white column), timolol-, (grey column) and latanoprost group (black column); *p*<*0.05, **p*<*0.01, ***p*<*0.001.

In both the subepithelial and deeper stroma, 30–40% of the area measured appeared as optically clear spaces, probably containing glycosaminoglycans, in control eyes, with a significant reduction in both medically treated groups. Subepithelially, the percentage area of empty spaces was significantly higher in latanoprost-treated specimens (19%) compared with timolol-treated eyes (13%) (p<0.001, fig 6, table 2).

## DISCUSSION

Several studies have already focused on the impact of long-term topical antiglaucoma medication on the histopathology of the conjunctiva and their possible effect on the outcome of filtering surgery. These studies reported a decrease in epithelial goblet cells,^2^ an increase in subepithelial collagen^3^ and increased amounts of macrophages, lymphocytes and mast cells in the substantia propria.^1^ ^10^ Moreover, topical antiglaucoma medication led to a significant degree of squamous metaplasia of conjunctival specimens^13^ and caused damage to the ocular surface.^14^ ^15^

The additional effect on these conjunctival changes by preservatives applied with the therapeutic agent has already been discussed in detail. BAC is the most commonly used preservative in eye-drops. In our study, the final BAC concentration applied within latanoprost and timolol was the same in sum (0.2 mg/ml) so that the conjunctiva in both groups was exposed to similar BAC effects. Interestingly, a recently published study by Liang *et al*^16^ showed that the new preservative-free tafluprost in contrast to latanoprost and BAC alone was less toxic in the rabbit conjunctica, indicating a good tolerability probably linked to the absence of preservative in the solution. In extrapolation, latanoprost induced less toxicity on the rabbit ocular surface than BAC. Pisella *et al* demonstrated in vitro and in vivo studies in which BAC-containing latanoprost and timolol exerted higher proinflammatory and proapoptotic effects on conjunctival cells than unpreserved substances.^17^ Preserved latanoprost, however, caused less toxicity than preserved timolol, and both drugs were less toxic than BAC alone.^17^ These results suggest a potential protective effect of the prostaglandin analogue and, to a lesser extent, of timolol against the toxicity of BAC in conjunctival cells. Guenoun *et al* assumed a protective effect of latanoprost against BAC toxicity probably being related to the antioxidative properties of latanoprost.^18^

In the present study the effect of latanoprost and timolol on the extracellular matrix (ECM) organisation, expression of matrix metalloproteinases (MMPs) and their inhibitors (TIMPs) on the human conjunctiva was investigated. MMPs form a group of proteolytic enzymes responsible for catalysing ECM degradation. TIMPs are involved in the maintenance of the ECM. The levels of MMPs, TIMPs and their isoforms have already been found in the ciliary body,^19^ aqueous humour,^20^ conjunctiva^21^ and optic-nerve head.^22^

An investigation of the TIMP level in aqueous humour detected an increase in TIMP-1 concentration in eyes with POAG in contrast to control eyes.^23^ Aqueous samples from these patients were also shown to increase collagen synthesis in vitro. According to these findings, the authors suspected that an increase in collagen synthesis and a decrease in collagen degradation may contribute to excessive deposition of collagen with loss of the trabecular cells during the development of POAG. Correspondingly, Ocklind *et al* observed in latanoprost-treated specimens of monkey eyes an increased expression of MMP-2 and MMP-3 and a decrease in collagen type IV and VI in the anterior part of the ciliary muscle^24^ suspecting that latanoprost-induced changes in the extracellular matrix might augment the flow of aqueous humour through the ciliary muscle bundles of the uveoscleral pathway. Additionally, Weinreb *et al* detected secretion of MMP-1 and MMP-9 in ciliary smooth muscle cells which was increased by application of prostaglandins.^25^

Increased levels of MMPs and TIMPs were also observed in diseases with an enhanced synthesis of ECM, like fibrotic disorders,^28^^–^31 proposing that MMPs and TIMPs are essential for the control of tissue remodelling after filtering surgery.^26^ Of note, MMPs and TIMPs were not found to be expressed in normal conjunctiva.^27^

In consistency with previously published data, in our study MMP-3 and MMP-1 expression was noted in the latanoprost treated conjunctival specimens but not in control eyes or timolol-reated eyes. The upregulation of MMPs might be based on the direct effect of prostaglandins. The increased expression of MMP-3, MMP-1, TIMP-1 and TIMP-2 in the latanoprost group may be related to the reduced collagen fibre density, indicating a direct effect of MMPs in terms of degrading the ECM. In contrast, in timolol-treated eyes, a very weak expression of MMPs and TIMPs was detected, but both the number of stromal and subepithelial collagen fibres and the amount of amorphous material, probably consisting of proteoglycans, were increased. Taking into account the study results of Mietz *et al*,^12^ a similiar MMP/TIMP-expression pattern was found in our study. Both the immunohistochemical staining and the transmission electron microscopy revealed remarkably similar results in the rabbit conjunctiva and the human conjunctiva.

In latanoprost-treated eyes, the amount of collagen fibres and amorphous material was lower, and the expression of MMPs and TIMPs was upregulated compared with the timolol group. As already reported, scarring and ECM accumulation within the subconjunctival space may be mainly responsible for filtering failure after trabeculectomy, suggesting that the stimulation of ECM degradation may suppress subconjunctival scar formation and promote longer survival of filtering blebs.^32^ Interestingly, electron microscopy of failed filtering blebs revealed a dense collagenous connective tissue as observed in our timolol-treated eyes. In contrast, in functioning blebs the subepithelial connective tissue was loosely arranged and contained histologically clear spaces,^33^ which was consistent with histological findings of our latanoprost-treated eyes. Of note, these clear spaces corresponded in size and position to microcystic spaces clinically.^33^ The clinical use of latanoprost for treatment of glaucoma might have two advantages. The main advantage of latanoprost is the potent efficiacy in lowering IOP. A second advantage might be the beneficial or less adverse effects that it has on the conjunctiva with respect to potential wound-healing processes following filtration surgery. Both factors seem to be related to each other. In this context, immunohistochemical data from Sagara *et al* revealed that intraocular pressure reduction which occurred with topical prostaglandin F2alpha was associated with a reduction in collagen within the uveoscleral outflow pathway in the anterior segment tissue of monkey eyes.^34^

In contrast, timolol-treated eyes showed an upregulation of CD 68 antibodies, which is an indicator for acute and chronic inflammatory infiltration. This observation suggests that long-term timolol treatment might be associated with a potentially higher risk for scarring processes around the scleral flap and a less successful long-term outcome after filtering surgery due to the altered histopathology.

In conclusion, our study data underlined that histomorphological changes were less pronounced in latanoprost-treated eyes compared with timolol-treated subjects, even in the human conjunctiva. The clinical relevance of the observed effects must be investigated in everyday practical experience especially with regard to the surgical outcome of filtering surgery after long-term use of timolol and latanoprost.
